# Speech connectedness predicts reading performance three months in advance: a longitudinal experiment

**DOI:** 10.1038/s41539-024-00248-4

**Published:** 2024-05-02

**Authors:** Bárbara Malcorra, Marina Ribeiro, Luísa Jensen, Giovana Gomes, Tamara Meletti, Natália Bezerra Mota

**Affiliations:** 1 Research department at Motrix, Motrix, Rio de Janeiro, Brazil; 2 Bioinformatics Multidisciplinary Environment (BioME), Federal University of Rio Grande do Norte (UFRN), Natal, Brazil; 3 Institute of Psychiatry (IPUB), Federal University of Rio de Janeiro (UFRJ), Rio de Janeiro, Brazil

**Keywords:** Education, Education

## Abstract

Aiming to verify the predictive value of oral narrative structure on reading acquisition, we followed 253 children (first and second graders) during an entire school year, assessing oral narratives and reading performances in five sessions. Transcriptions of oral narratives were represented as word-recurrence graphs to measure connectedness attributes. Connectedness predicted performance in phonological awareness, reading comprehension, and word reading accuracy 3–4 months in advance.

## Main

Learning to read promotes a true cognitive revolution during elementary school. Reading is the basis of educational communication for other disciplines that require language abilities (such as history, geography, sciences, and others). Also, by mastering grammar and syntax rules, the learner can access more structurally complex content as advances in learning. The increase in language structure complexity from oral narratives is also related to signs of a more complex mental organization informing a child’s cognitive development^1^. Based on the idea that how people tell stories reveals how they organize their thoughts, a graph-theoretical-based approach - in which each node represents a word, and each directed edge represents the temporal order of consecutive words - is quite informative in naturalistic settings in different contexts^1^.

Previous studies have reported that the oral narrative structure changes from a short to a long-range recurrence pattern as soon as a child starts to read, increasing connectedness^2^. In Mota et al.^3^, narratives from seventy-six second-grade children (ages from 6 to 8 years old) were collected in the middle of the school year, together with the intelligence quotient (IQ), theory of mind (ToM), as well as reading and math scores acquired from a national assessment four months later. The results reveal that the Largest Strongly Connected component (LSC) predicted the national reading exam performance five months later. Furthermore, the results reveal that the association with reading performance was independent of IQ and ToM performances. A subsequent study with the same participants but in the third grade reported that exclusively verbal short-term memory was associated with connectedness measures (LSC). In addition, the correlation with reading fluency was significant only in the second grade, pointing to possible dynamics associated with the reading acquisition process^4^.

Although promising, the previous studies did not detail which reading acquisition aspects of this sign of oral language reflect written language acquisition. Does orality structure relate to initial steps such as phonological awareness? In the same way, does it relate to the ability to correctly read words, until sentences, and small texts, considering reading fluency and comprehension? In a longitudinal assessment, the present study aims to investigate these associations (how narrative structure relates to different reading performances) and to further explore the potential of graph connectedness as a tool to predict future reading difficulties in advance. First, we aim to verify the association between graph connectedness and reading performance over the school year, during the literacy period, following your development. Second, to verify the predictive value of graph connectedness concerning reading performance tests months in advance.

**Fig. 1:**
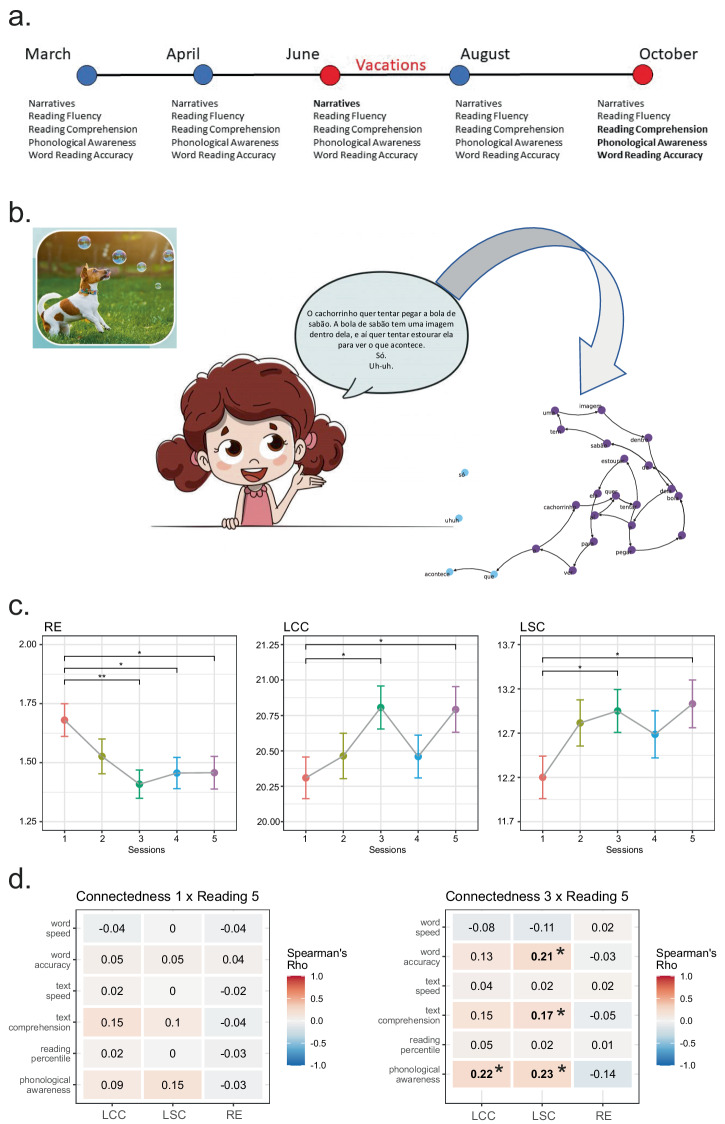
Longitudinal changes of word recurrence graphs associated with reading acquisition. **a** Longitudinal assessment during an entire school year considering vacations. Red dots highlight the measurements associated with time (marked in bold). Blue dots are the other data collection points. **b** Word recurrence methodological example, from a text (storytelling based on an affective picture) to a graph representation. The oral narrative is transcribed, each word is represented as a node, and directed edges represent the word sequence. **c** Connectedness differences occurring during the entire school year assessment. Pairwise significant differences marked with *. **d** Correlation matrix between word recurrence graph connectedness (collected at the first or the third session) and reading abilities. Significant results after multiple comparison corrections in bold and marked with *.

To do so, five assessments were performed over the year 2022. The first one was performed in March, at the beginning of the school year. Four subsequent assessments were performed in April and June, right before school vacations, and August and October (Fig. 1a). The longitudinal design of this study allowed us to investigate the relationships between graph attributes and reading performance during the school year, as well as their development curves. Based on previous studies that revealed a complex association between speech connectedness and reading skills^2–4^, we hypothesized that (1) higher connectedness would be associated with better reading performance over the school year and (2) measurements of oral narrative graphs would predict reading performance months in advance.

Comparisons between the five assessments made during the school year revealed differences in the three graph connectedness attributes (Kruskal–Wallis test; *p*-values < 0.05). Pairwise comparisons revealed significant differences between the first (March) and third (June) assessments, as well as the first and the fifth (October) assessments in all three graph attributes (Fig. 1c). As short-range recurrences (RE) decrease, connectedness (Largest Connected Component (LCC) and LSC) increases, with an interesting interruption of this dynamic between the third and the fourth measures (related to vacations in the middle of this school year). This result points to an effect related to the school environment.

Given these results, we studied the associations between oral narratives collected in March or June, predicting reading performance in October. Significant Spearman positive correlations between reading performance and LCC and LSC were found. Connectedness from oral narratives in June (third assessment) positively correlated with phonological awareness, reading comprehension, and word accuracy taken in October (fifth assessment) (Fig. 1d).

The structural analyses based on word recurrence graphs here allowed us to replicate in a larger sample from a longitudinal experiment the dynamic changes in oral narratives during reading acquisition: as short-range recurrence (RE) drops, connectedness increases with an essential effect of the school environment. The measures from a single point obtained from 3 different stories are also stable (Supplementary Fig. 1). Interestingly, we found a higher connectedness for girls than boys and a diverse pattern of association with reading performances that should be addressed in future studies (Supplementary Note 1). Moreover, the oral narratives connectedness was predictive 3–4 months in advance of reading abilities related to phonological awareness, reading accuracy, and text comprehension, but not reading fluency. The reading acquisition that happened at the beginning of the school year, before vacations, mirrors the development after returning to the school environment.

These results point to a feasible, low-cost, and inclusive assessment based on storytelling (as it does not require the child to be able to read), enabling the tracking of aspects of cognitive development related to reading acquisition on a large scale. For Latin American countries such as Brazil, scalable solutions could be crucial to planning public educational policies based on data.

Future studies should examine the relationship between reading and language connectivity in a more diverse sample, including not only families with high socioeconomic status and educational levels, but also less privileged families with low socioeconomic status and educational levels (a scenario that is still a reality in developing countries like Brazil), and examine whether it would be possible to replicate the results in a more heterogeneous sample.

In addition, future studies could evaluate other measures based not only on transcriptions but also on acoustic signals and facial expressions, which could help us understand communication in depth, as well as the role of a child’s temperament, considering that the ability to speak may be challenging for shy children.

## Methods

### Participants

A total of 253 children (119 females and 134 males, aged 5–8 years, 6.27 ± 0.62, mean ± SD) were recruited from three private schools in São Paulo, Brazil. The participants were from the first and second school years. Exclusion criteria include (1) children with psychiatric or neurological disorders and (2) children using psychotropic medication. The children came from families with high levels of educational attainment and high socioeconomic status (family monthly income R$ 22,500.00 ± 24.9).

The Ethics on Research Committee of the Federal University of Rio Grande do Norte (UFRN), Brazil, approved the study under report number 5.201.989, CAAE registry number 53538521.5.0000.5263. The data were collected during regular class hours within the school setting. Each child was evaluated individually in a classroom assigned exclusively for this purpose. All the protocols were previously explained at a meeting between experimenters, legal guardians, and teachers. Written informed consent was obtained from all children’s legal guardians at the beginning of the experiment. The author (BM) interviewed the legal guardians, who collected information regarding the socioeconomic questionnaire and the children’s health conditions.

### Protocol

Longitudinal data regarding oral narrative recordings and reading performance tasks were collected at five-time points comprising the whole school year of elementary school’s first and second grades (March, April, June, August, and October, Fig. 1a). Each session lasted about 15 min per child on average.

### Storytelling task

The children were presented with three images of positive affective content (a baby, a dog, and a dessert), content chosen from the IAPS database^5^. The images were shown to the participants on a computer screen. The interviewer explained the procedure previously. The children were instructed to pay attention to each of the images for 15 s and then report three narratives (one for each image) on the content of the images. After seeing each image for 15 s, the computer screen was turned off, and the children were asked to report a narrative regarding what was happening in that image^4^. To minimize differences between participants in the reports, each narrative was limited to 30 s^4^. If the children did not reach the minimum time, the interviewer gave a general stimulus such as “Could you talk more about it?”. The pictures were unavailable for the participants while the story was being told. Speech samples were audio-recorded, transcriptions were made by three authors, and discrepancies between the three were resolved through discussion.

### Reading performance tasks

Three assessments of reading performance were administered - namely (a) eye-tracking text reading task, (b) single-word reading task, and (c) phonological awareness task - as described below.

In the eye-tracking text reading task, children performed a reading task while their eye movements were recorded using a Tobii screen-based eye tracker with a 120 Hz sampling frequency. Physiological tests based on eye tracking were applied during three reading activities^6^. In the first activity, participants must name a sequence of letters (Rapid automated naming - RAN). In the second and third activities, participants must read two passages of text aloud, appropriate for age and school grade, and then answer six comprehension questions, three after each text. The reading speed average was assessed from this task. Several eye movement parameters were computed using the eye tracking data from the whole passage reading task, such as the average speed reading, reading percentile, and reading comprehension. Although we used eye-tracking, we could not access the raw eye-tracking data. We only had access to the measurements collected and made available in an automated way by Lexplore’s solution.

In the single word reading task, children’s reading fluency was measured. The task consisted of a list of 32 words, in which children were asked to read as fast as possible. Word accuracy and time of reading (word speed) were measured. Word accuracy was calculated with the formula: (*hits*/*trials*)*(100*trials)/32, where *hits* are equivalent to the total number of words read correctly, and *trials* are equivalent to the number of trials the child made. Word speed was calculated with the formula: time*32/*trials*, where *time* is equivalent to the seconds taken to read the entire list of words, and *trials* are equivalent to the number of trials the child made. There was no time limit for the task.

Phonological awareness abilities were investigated using the Phonological Awareness: Instrument of Sequential Assessment^7^, which involves five subtests that assess initial syllable identification, rhyme, initial phoneme, final phoneme, and transposition. Two training items are initially applied in all subtests and then the evaluation items. The maximum score was 20 correct answers, with a minimum score of 4 correct answers for each subtest. There was no time limit for the task.

### Graph analysis procedure

Since the comparison between the three images used to collect oral narratives (a baby, a dog, and a dessert) did not show significant differences regarding the number of words (Supplementary Fig. 1), we concatenated the three narratives into one, adding a line break between them. After, a moving window of a fixed word length (30 words) with a step of one word was performed to control the verbosity effect. The average graph attributes of all graphs of 30 words were calculated and used for statistical analysis. Based on previous findings, we focused on the following three connectedness attributes: (1) repeated edges (RE), defined as the sum of all edges linking the same pair of nodes; (2) the number of nodes in the largest connected component (LCC), defined as the largest set of nodes directly or indirectly linked by some path; and (3) the number of nodes in the largest strongly connected component (LSC), defined as the largest set of nodes directly or indirectly linked by reciprocal paths, so that all the nodes in the component are mutually reachable^1,8,9^.

Adopting the method used here (in which each word of a narrative is represented as a node, and the sequence between words is represented as direct edges), we characterize the relationship between the nodes (words) in the phenomenon (narrative) that determines this sequence, which means that word recurrences determine topological metrics, from short-range recurrences (such as repeated edges, which are repetitions of the same word association) to long-range recurrences or connectivity (such as the number of words [nodes] connected in a single component, LCC and LSC). Just as the sequence of words defines the relationship between nodes, rather than semantic content or linguistic relationships, the pattern of word recurrence defines the topological metrics. Therefore, these graphs are not based on meaning but on recurrence patterns. It is vital to understand that word recurrence graphs can reveal patterns of connectedness from the earliest stages of language acquisition because they do not rely on meaning or linguistic rules.

When a word is repeated in the story, it returns to the same node initially used, closing larger or smaller cycles (recurrences), which characterizes these recurrences (short and long) and allows for increasingly complex connectivity. In this sense, the reduction of RE (repeated edges) and the increase of LCC and LSC may represent a story with few repetitions of the same word association (or word associations) and highly connected complex components with a larger lexical diversity (in the case of LCC increase); in a reciprocal manner (in the case of LSC increase).

Previous studies have identified developmental patterns of these measures across the lifespan: during childhood and at formal education exposure, as child ages and advances in schooling, there is an increase of lexical diversity (LCC) and reciprocal recurrence connectedness (LSC), while repetitions of the exact words associations decrease; during aging in adulthood, as we age, our speech becomes more repetitive (characterized by an increase in short recurrences such as repeated edges) and less connected (characterized by a decrease in longer recurrences as LCC and LSC).

### Scalability

Our protocol provides a safe and scalable way to evaluate public educational policies. We have created a narrative collection application that displays three images with positive content, such as a baby, a dog, and a treat. Participants are asked to focus on each image for fifteen seconds and then create a narrative for each one. Once all three narratives are complete, the stories are recorded and transcribed automatically using an AI-based algorithm. Each narrative is represented computationally by a word recurrence graph, which is analyzed to determine academic development.

In a typical school environment (without signs/symptoms of mental distress), graph attributes indicate academic development, according to Mota et al.^1,2^. Therefore, our protocol can be a valuable, language-independent tool for education professionals. As a monitoring tool, we can plot the information on the narrative structure obtained through graphs on a normative curve representing the evolution of oral narrative complexity throughout the school years. With this curve, we can evaluate the development of the user’s communication skills compared to their peers in the same or different school years, providing a global perspective of their ability to generate narratives.

### Statistical analyses

First, we checked whether there was a normal distribution for the graph attributes using the Shapiro–Wilk test (Supplementary Table 1). Since the data were not normally distributed (*p* < 0.01), we used a nonparametric Kruskal–Wallis test. We computed the effect size for the Kruskal–Wallis test as the eta squared based on the H-statistic: *eta2[H]* = *(H* *−* *k* + *1)/(n* *−* *k)*, where *H* is the value obtained in the Kruskal–Wallis test; *k* is the number of groups; *n* is the total number of observations^10^ (Supplementary Table 2), followed by pairwise comparisons Wilcox test, to verify if there was a change in the complexity pattern over the five assessments (correction for two comparisons, alpha = 0.025; Supplementary Table 3). Second, Spearman’s correlations were used to investigate the relationship between graph attributes and reading performance (word accuracy, phonological awareness, comprehension, reading speed average on texts, total time in the word reading task, and reading percentile) (correction for two comparisons, alpha = 0.025; Supplementary Table 4). Correction for multiple comparisons using the Bonferroni method included six comparisons (correct α = 0.0083). All the analyses were performed in RStudio 4.1.0^11^.

### Supplementary information

Supplementary information

## Data Availability

The datasets used and/or analyzed during the current study are available from the corresponding author upon reasonable request.
