# Supplementary material for: Speech connectedness predicts reading performance three months in advance: a longitudinal experiment
Source: NPJ Sci Learn. 2024 May 2;9:35. doi: 10.1038/s41539-024-00248-4 (PMC11063169; doi:10.1038/s41539-024-00248-4)
Supplement: Supplementary file 1 — Supplementary information [file 41539_2024_248_MOESM1_ESM.pdf]

Supplementary information

Supplementary Table 1: Descriptive table of speech connectedness and reading performance

Descriptive table	**Mean**	**SD**	**Min**	**Max**	**p-value (Shapiro-Wilk)**	**Sample size**
Word speed (1ª session - March)	316.92	361.59	26	3096	1.44792E-18	195
Word speed (3ª session - June)	202.8	251.66	18	1620	1.09725E-17	161
Word speed (5ª session - October)	199.68	247.27	18	1620	3.8179E-19	205
Word accuracy (1ª session - March)	54.81	40.43	0	100	3.3077E-15	195
Word accuracy (3ª session - June)	70.7	34.1	0	100	5.06678E-15	162
Word accuracy (5ª session - October)	69.73	36.24	0	100	2.76326E-17	205
Text speed (1ª session - March)	50.63	31.64	4.5	132	8.9333E-05	123
Text speed (3ª session - June)	54.49	32.8	7.5	137	0.000203634	138
Text speed (5ª session - October)	58.86	36.93	4	158	9.10806E-05	167
Text comprehension (1ª session - March)	3.43	1.96	0	6	6.47999E-13	248
Text comprehension (3ª session - June)	4.17	1.86	0	6	1.35871E-14	226
Text comprehension (5ª session - October)	4.46	1.85	0	6	2.81799E-16	217
Reading percentile (1ª session - March)	76.37	27.38	3	100	3.41151E-11	123
Reading percentile (3ª session - June)	77.95	26.2	3	100	4.06944E-12	138
Reading percentile (5ª session - October)	73.69	31.53	3	100	4.1256E-14	167
Phonological awareness (1ª session - March)	13.3	4.36	0	20	3.32704E-07	249
Phonological awareness (3ª session - June)	14.82	3.88	2	20	1.07602E-08	228
Phonological awareness (5ª session - October)	15.51	3.62	4	20	4.6792E-09	221
LCC (1ª session - March)	20.31	2.31	11.25	24.5	4.1186E-09	240
LCC (3ª session - June)	20.81	2.29	7.85	25.6	4.40963E-12	217
LCC (5ª session - October)	20.79	2.4	6	24.97	1.10116E-12	205
LSC (1ª session - March)	12.2	3.75	1	18.86	6.62361E-08	240
LSC (3ª session - June)	12.91	3.7	2	18.36	4.88833E-09	217
LSC (5ª session - October)	13.03	4.01	1	18.41	7.29848E-12	205
RE (1ª session - March)	1.68	1.09	0	8.41	4.56739E-15	240
RE (3ª session - June)	1.41	0.9	0	6	1.02411E-08	217
RE (5ª session - October)	1.46	1.03	0	6.33	3.1293E-11	205
Age (years)	6.27	0.63	5	8	8.97357E-18	249

Legend: SD = standard deviation; range given from minimum (Min) and maximum (Max) values, p-values from normality test (Shapiro-Wilk test), and sample size.

Supplementary Table 2: Kruskal-Wallis test

variable	n	statistic	p	effect size	magnitude	p session 1-3*	p session 1-5*
LCC	1160	11.45979	**0.0219**	0.006459	small	**0.015**	**0.008**
LSC	1160	11.41734	**0.0223**	0.006422	small	**0.013**	**0.001**
RE	1160	10.52708	**0.0324**	0.005651	small	**0.005**	**0.013**

Legend: Significant p-values from the Kruskal-Wallis test are indicated in bold. The effect size for the Kruskal-Wallis test was calculated as the eta squared based on the H-statistic: eta2[H] = (H - k + 1)/(n - k); small effect: 0.01- < 0.06; moderate effect: 0.06 - < 0.14; large effect: >= 0.14 (Tomczak & Tomczak, 2014).
* Pairwise comparisons Wilcoxon test with significant p-values indicated in bold. Correction for two comparisons: alpha = 0.025.

Supplementary Table 3: Pairwise comparisons Wilcoxon test

var1	var2	n1	n2	statistic	p
LCC_1	LCC_2	237	223	25179.5	0.382
LCC_1	LCC_3	237	216	22195	**0.015**
LCC_1	LCC_4	237	206	23840	0.671
LCC_1	LCC_5	237	205	20741	**0.008**
LCC_2	LCC_3	223	216	21907	0.101
LCC_2	LCC_4	223	206	23453	0.706
LCC_2	LCC_5	223	205	20608	0.078
LCC_3	LCC_4	216	206	24785	0.043
LCC_3	LCC_5	216	205	21738	0.748
LCC_4	LCC_5	206	205	18551.5	0.033
LSC_1	LSC_2	237	223	23245.5	0.026
LSC_1	LSC_3	237	216	22143.5	**0.013**
LSC_1	LSC_4	237	206	21804	0.052
LSC_1	LSC_5	237	205	19946	**0.001**
LSC_2	LSC_3	223	216	24078	0.997
LSC_2	LSC_4	223	206	23437.5	0.715
LSC_2	LSC_5	223	205	21955.5	0.481
LSC_3	LSC_4	216	206	22845	0.634
LSC_3	LSC_5	216	205	21376	0.541
LSC_4	LSC_5	206	205	19794	0.273
RE_1	RE_2	237	223	29168	0.054
RE_1	RE_3	237	216	29516.5	**0.005**
RE_1	RE_4	237	206	27820.5	**0.011**
RE_1	RE_5	237	205	27629.5	**0.013**
RE_2	RE_3	223	216	25044.5	0.47
RE_2	RE_4	223	206	23619	0.613
RE_2	RE_5	223	205	23631.5	0.545
RE_3	RE_4	216	206	21908	0.786
RE_3	RE_5	216	205	21962	0.887
RE_4	RE_5	206	205	21274.5	0.895

Legend: Pairwise comparisons Wilcoxon test with significant original p-values indicated in bold. Correction for two comparisons: alpha = 0.025.

Supplementary Table 4: Spearman correlations between graph attributes and reading performance

graph attributes (3ª session - June)	reading performance (5ª session - October)	Rho	p
LCC	reading percentile	0.05234	0.509641
LSC	reading percentile	0.021737	0.784321
RE	reading percentile	0.011816	0.881738
LCC	text comprehension	0.145696	0.035299
LSC	text comprehension	0.174148	**0.011674**
RE	text comprehension	-0.05335	0.442977
LCC	text speed	0.040789	0.607436
LSC	text speed	0.019874	0.80241
RE	text speed	0.024147	0.761088
LCC	word accuracy	0.126769	0.076634
LSC	word accuracy	0.206262	**0.003726**
RE	word accuracy	-0.02539	0.723951
LCC	phonological awareness	0.219564	**0.001365**
LSC	phonological awareness	0.225576	**0.000995**
RE	phonological awareness	-0.14084	0.041455
LCC	word speed	-0.08416	0.240906
LSC	word speed	-0.10592	0.139526
RE	word speed	0.018581	0.796029

Legend: Spearman correlations with significant p-values are indicated in bold. Correction for two comparisons: alpha = 0.025.

Supplementary Figure 1: Original graph measures show no difference between the three stimulus

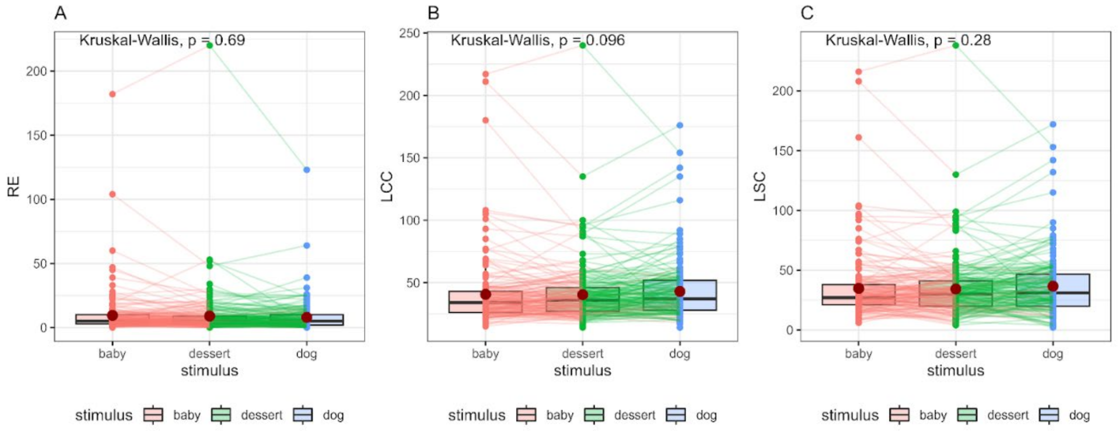

Legend: Red dots represent the average of graph attributes in each stimulus.

Supplementary Note 1: Differences between girls and boys: a complementary analysis

Several studies have indicated an advantage for girls over boys in various aspects of language development, such as word comprehension and production, particularly in the early stages of lexical development (e.g., Bornstein et al., 2004; Fenson et al., 2007; Galsworthy et al., 2000).

Here, we compared the graph attributes between boys and girls in the five sessions and found significant differences in LCC and LSC ($p < 0.05$), as shown in the figure below, which is probably because girls had a larger expressive vocabulary size than boys (Rinaldi et al. 2021).

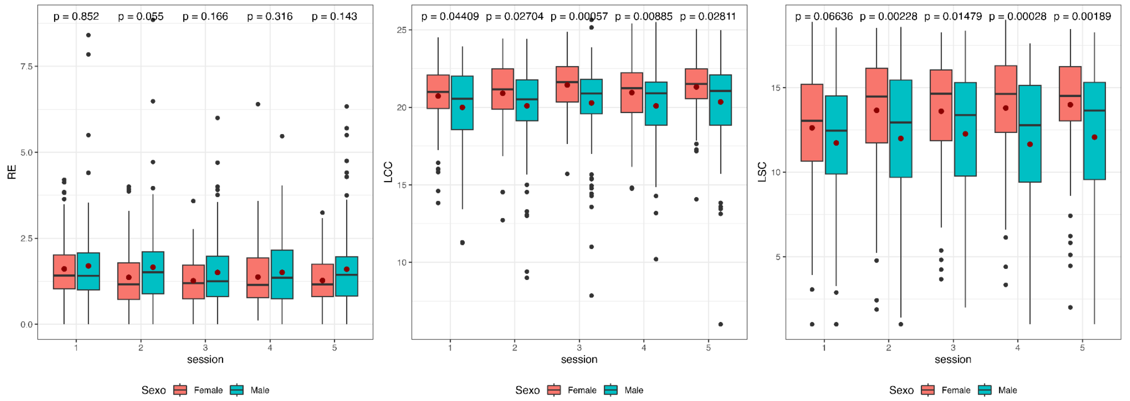

Legend: Comparison of the connectedness attributes between boys and girls in the five assessments. Red dots represent the average of graph attributes in each stimulus.

In addition, considering boys and girls, we verified a diverse pattern of associations between oral narratives collected in March or June, predicting reading performance in October. Significant Spearman positive correlations between word accuracy and LCC (Rho = 0.23) and LSC (Rho = 0.29) from oral narratives in June (third assessment) were found for girls only. Moreover, connectedness positively correlated with phonological awareness in October (fifth assessment; Rho = 0.30). For boys, connectedness from oral narratives in June (third assessment) positively correlated with phonological awareness only (Rho = 0.28). Also, only for boys, there was a negative correlation between phonological awareness and RE (Rho = -0.22). A correlation between narrative connectedness and reading comprehension was found when combining boys and girls exclusively (girls isolated showed larger effect sizes but insignificant).

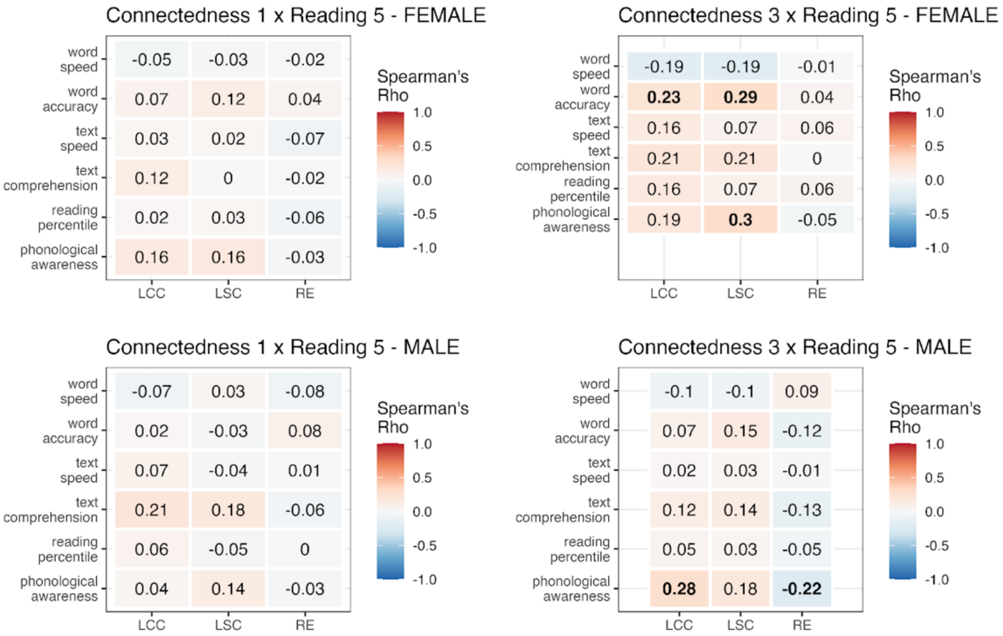

Legend: Correlation matrix between word recurrence graph connectedness (collected at the first or the third session) and reading abilities for boys and girls separately. Significant results after multiple comparison corrections in bold.

Given the small effect sizes and the limited number of participants, more extensive studies should be planned to understand this diversity better, especially considering the different stages of lexical development between boys and girls.

References

Bornstein, M. H., Hahn, C.-S., & Haynes, O. M. (2004). Specific and general language performance across early childhood: Stability and gender considerations. First Language, 24(3), 267–304. https://doi. org/10.1177/0142723704045681

Fenson, L., Dale, P. S., Reznick, J. S., Bates, E., Thal, D. J., Pethick, S. J., Tomasello, M., Mervis, C. B., & Stiles, J. (1994). Variability in early communicative development [Monograph]. Society for Research Child Development, 59, 1–189. https://doi.org/10.2307/1166093

Galsworthy, M. J., Dionne, G., Dale, P. S., & Plomin, R. (2000). Sex differences in early verbal and non-verbal cognitive development. Developmental Science, 3, 206–215. https://doi.org/10.1111/1467- 7687.00114

Rinaldi, P., Pasqualetti, P., Volterra, V., & Caselli, M. C. (2023). Gender differences in early stages of language development. Some evidence and possible explanations. Journal of Neuroscience Research, 101, 643–653. https://doi.org/10.1002/jnr.24914
